# Gastrodin Promotes the Survival of Random-Pattern Skin Flaps via Autophagy Flux Stimulation

**DOI:** 10.1155/2021/6611668

**Published:** 2021-01-09

**Authors:** Hongyu Chen, Baoxia Chen, Baolong Li, Xiaobin Luo, Hongqiang Wu, Chenxi Zhang, Junling Liu, Jingtao Jiang, Bin Zhao

**Affiliations:** ^1^Department of Orthopedics (Division of Plastic and Hand Surgery), The Second Affiliated Hospital and Yuying Children's Hospital of Wenzhou Medical University, Key Laboratory of Orthpedics of Zhejiang Province, The Second School of Medicine, Wenzhou Medical University, Wenzhou 325027, China; ^2^Department of Postanaesthesia Care Unit, The Second Affiliated Hospital and Yuying Children's Hospital of Wenzhou Medical University, The Second School of Medicine, Wenzhou Medical University, Wenzhou 325027, China

## Abstract

The random-pattern flap has a significant application in full mouth restoration (reconstructive surgery) and plastic surgery owing to an easy operation with no axial vascular restriction. However, distal necrosis after flap operation is still considered the most common complication which makes it the Achilles heel in the clinical application of random-pattern flaps. A Chinese medicinal herb named gastrodin is an effective active ingredient of *Gastrodia*. Herein, the existing study explored the significant potential of gastrodin on flap survival and its underlying mechanism. Our obtained results show that gastrodin will significantly improve flap survival, reduce tissue edema, and increase blood flow. Furthermore, our studies reveal that gastrodin can promote angiogenesis and reduce the apoptotic process as well as oxidative stress. The results of immunohistochemistry and immunoblotting revealed that gastrodin has a role in the elevation of autophagy flux which results in induced autophagy. The use of 3MA (3-methyladenine) for the inhibition of induced autophagy significantly weakened the underlying benefits of gastrodin treatment. Taken together, our obtained results confirmed that gastrodin is an effective drug that can considerably promote the survival rate of flaps (random pattern) via enhancing autophagy. Enhanced autophagy is correlated with the elevation of angiogenesis, reduced level of oxidative stress, and inhibition of cell apoptosis.

## 1. Introduction

Skin flap transfer is a form of transplantation that is widely used in plastic and reconstructive surgery. In clinical settings, random-pattern skin flaps have become one of the most considerably and highly used techniques to cure multiple defects in tissues. It is mostly used for the reconstruction of skin defects that are correlated with congenital diseases, trauma, diabetes, and cancer-related abnormalities [1, 2] [3], although blood circulation is not specific in flaps (random-pattern) and is mostly associated with the development of flap necrosis after surgery. In the reconstruction process, the underlying postoperative complication is still challenging [4]. The length-to-width ratio is restricted to 1.5 or 2, which limits the clinical application of random flaps [5]. In the flaps, the supply of blood is sustained by the vascular network in the flap pedicle bed [6]. As the random flaps are formed without retaining the vascular pedicle, significant ischemia occurs, and then angiogenesis begins at the flap pedicle. So, angiogenesis has been very low at the flap distal end, which leads to tissue necrosis in the underlying area that is mostly caused by insufficient angiogenesis [7]. In addition, post neovascularization, the recovery and blood reperfusion enhance the IRI of the ischemic skin flap. Numerous studies have revealed that oxidative stress and the apoptotic process are the two main processes of IRI. Besides, the underlying processes are the significant factors of random flap necrosis [8, 9]. Considering the underlying mechanisms, in view of the widespread use of random flaps in clinical practice and the high rate of flap necrosis, the potential approaches that enhance angiogenesis and decrease oxidative stress and cellular apoptosis have been well studied in recent decades. Different approaches have been used to enhance the flap survival rate [10, 11]. At present, vascular and surgical delays are the only practical methods that are considered to be effective in improving the survival rate of skin flaps, although these methods are time consuming and expensive [12]. Taken together, medical treatments should be emphasized to improve flap survival.

Autophagy is a cellular degradation process and has a protective role in the maintenance of cellular homeostasis by enhancing the survival and the functions of the cell [13]. In the underlying process, the variations occur in the subcellular membrane structure. Then, the cytoplasmic components are wrapped, resulting in the formation of autophagosomes, and finally, the degradation of cell contents occur. The underlying process is known as autophagic flux and has been regarded as a significant indicator of autophagy activity [14]. Many studies have revealed that autophagy significantly contributes to enhancing angiogenesis, decreasing oxidative stress, and attenuating the apoptotic process [15, 16]. The stimulation of autophagy enhances the angiogenesis of bovine aortic endothelial cells (ECs). The generation of ROS and the stimulation of AKT play a key role in the process of angiogenesis [17]. Current studies have revealed that during oxidative stress, autophagy significantly contributes to the degeneration of the intervertebral disc by decreasing mitochondrial dysfunction as well as apoptosis [18]. In addition, the upregulation of autophagy can reduce IRI and inhibit a high level of apoptosis activated via intestinal IRI [19]. Calcitriol remarkably improves random flap sustainability via stimulating autophagy that can relieve IRI and enhance angiogenesis [5, 20]. It also attenuates oxidative stress as well as apoptosis that results in enhancing the random flap survival rates.

Gastrodin (GAS) is a phenolic glycoside which is soluble in water and has a significant oral bioavailability. It has been isolated from the TCM, i.e., *Gastrodia elata* [21]. Gastrodin is the active ingredient of *Gastrodia* and has reported activity against inflammation [22]. It also possesses antioxidant [23], antinociceptive [24], and antiapoptotic effects in many diseases [25]. According to reports, gastrodin reduces the expression of proinflammatory factors to reduce lipopolysaccharide-induced acute lung injury [26]. GAS protects human dopaminergic cells from oxidative stress via stimulating the Nrf2/HO1 pathway cascade [27]. Besides, gastrodin reduces cerebral ischemic damage in rats by inhibiting inflammatory response and apoptosis [28]. Moreover, its function in angiogenesis and in the survival of random flaps is still elusive, and no study has been reported on the role of gastrodin in random flaps. The current study is aimed at studying the role and mechanism of gastrodin in random flaps. Herein, we speculate that gastrodin may activate autophagy flux to reduce oxidative stress, promote angiogenesis, inhibit cellular apoptosis, and improve the survival of random flaps.

## 2. Materials and Methods

### 2.1. Animals and Groups

Eighty-four healthy Sprague-Dawley rats (male: 250-300 g) were purchased from the Experimental Animal Center of Wenzhou Medical University (authorization number: (ZJ) 2005-0019). Guidance for *in vivo* studies was provided via the “Guidelines for the Care and Use of Laboratory Animals” of the Chinese Institutes of Health. Animal experiments, breeding, surgery, and final execution were conducted. All rats were individually kept in highly maintained experimental cages with a 12 hr light and 12 hr dark duration. Normal food and water were freely available during any experimental procedures. Here in the existing study, the rats were randomly classified into four groups, i.e., control (*n* = 24), gastrodin (GAS, *n* = 24), gastrodin and 3-methyladenine (GAS+3MA, *n* = 18), and 3-methyladenine (3MA, *n* = 18).

### 2.2. Reagents and Antibodies

GAS (C13H18O7; purity ≥ 98%) and 3-methyl adenine (3MA) (C6H7N5; purity ≥ 98%) were provided by Sigma-Aldrich, USA. Pentobarbital sodium, diaminobenzidine (DAB) developer, lead oxide-gelatin, and staining kit, i.e., H&E, were procured from Beijing SolarBio Science & Technology Co., Ltd. (China). Anti-cadherin 5 monoclonal antibodies and anti-GAPDH monoclonal antibodies were procured from Wuhan Boster Biological Technology, Ltd. (Wuhan, China) and Bioget Technology (Shanghai, China) accordingly. VEGF, SOD1, MMP9, anti-HO1, and anti-CASP3 monoclonal antibodies were procured from the Proteintech Group (Chicago). eNOS, CYC, and anti-Bax (Bax) monoclonal antibodies were obtained from Cell Signaling Technologies (Beverly, Massachusetts). Anti-SQSTM1/p62 monoclonal antibodies were provided by Abcam (Cambridge, UK). The monoclonal antibodies, i.e., LC3 and 3MA were procured from Sigma-Aldrich, USA. HRP-labeled IgG secondary antibodies were procured from Santa Cruz Biotechnology Inc. (Dallas, Texas, USA). FITC-labeled IgG secondary antibodies and DAPI solutions were provided by Boyun Biotechnology (Nanjing) and Jiangsu Beyotime, respectively. The BCA kits and the ECL Plus Reagent Kits were obtained from Thermo Fisher Scientific and PerkinElmer Life Sciences accordingly.

### 2.3. Drug Administration

In previous studies, the use of 2 percent DMSO (in physiological saline) as a solvent for intraperitoneal injection in rats proved to be safe and did not produce obvious toxicity [6]. Therefore, the GAS solution was prepared in 2 percent DMSO in physiological saline (25 mg/mL concentration). After surgery for 7 days consecutively, GAS (25 mg per kg of body weight, daily) was given to all rats by IP injections in the GAS group. The GAS+3MA group was exposed to 3MA (15 mg per kg of body weight) 30 minutes before each GAS administration. The control group (solvent control) was exposed to an equal volume of DMSO physiological saline solution, while the 3MA group was exposed to the same volume of 3MA (15 mg per kg of body weight) for 7 days. All rats were sacrificed with sodium pentobarbital (overdose), and collection of histological specimens followed.

### 2.4. Random-Pattern Skin Flap Model

**Figure 1:**
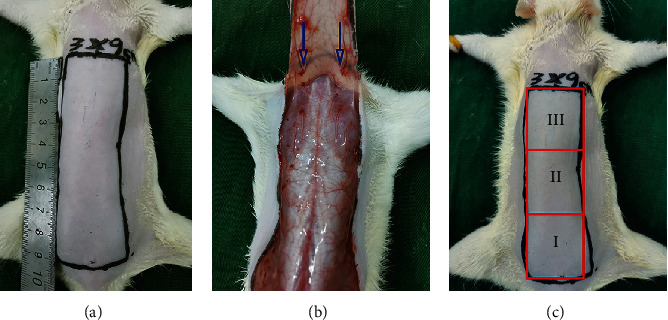
The design of the random-pattern flap model. (a) Marking a 3 × 9 cm^2^ flap on the dorsal area of the rat. (b) The tail-based skin/aponeurotic flap (size 3 × 9 cm^2^) is lifted to the back of the rat under the fascia, and the left and right sacral arteries that support the supply of the blood in the flap are entirely removed. (c) The area of the random flap area has been classified into proximal (area I), middle (area II), and distal (area III) areas. All areas are of equal size.

Prior to surgery, the anesthetization of rats was carried out via an IP injection of 2 percent (*w*/*v*) sodium pentobarbital (40 mg per kg of body weight). According to the reported studies, a developed “McFarlane flap” model of the rat's back was prepared (each rat was at the same position) [29]. The random flap is created by incising the skin along the designated line (3 × 9 cm). All known blood vessels under the flap were completely cut off before raising the double-pedicle flap (Figures 1(a) and 1(b)). Finally, intermittently use 4-0 sutures to suture the flap back to its original position. Three independent equal areas are divided on each flap: area I (relative to the proximal base of the tail of the flap), area II, and area III (most distal) (Figure 1(c)).

### 2.5. General Evaluation of Flap Survival

On the third and seventh day postoperation (POD), the survival of the flap was assessed by high-quality photography. Macroscopic changes of the flap, i.e., hair condition, color, and texture, were observed. The survival area was found to be pink, soft-skinned, and with new hair growth. The necrotic area was found to have scabs, hardened and dark lesions, and no new hair growth. All photos were measured with ImageJ software (MD, United States). For the calculation of the survival area, the survival area percentage was measured as follows: survival area range/total area × 100%.

### 2.6. Measurement of Tissue Edema

Tissue edema is a key factor that results in ischemic flap necrosis. Therefore, the degree of edema is a significant indicator for evaluating necrosis and was determined by evaluating the water content in the skin flap. Skin flap specimens (*n* = 6) were taken randomly, followed by their dehydration in a 50°C autoclave at the 7th POD. The sample was weighed every day until a constant weight was obtained for more than 48 h. The moisture content was measured as follows: ([wet weight − dry weight] ÷ initial weight)100%.

### 2.7. Laser Doppler Blood Flow Imaging

Laser Doppler Blood Flow (LDBF) is an image of blood vessels under the tissue surface, which can improve the display of small blood vessels under the tissue surface. The LDBF imaging was used to determine the vascular flow as well as the blood supply of the flap [30], which was the perfect choice for evaluating angiogenesis. The LDBF measurement scheme has been described previously [31]. A rat under anesthesia was placed in the prone position in the scanning area, and the laser Doppler imager was employed for the scanning of the entire area of the skin flap. The images of blood flow on 0, 3rd, and 7th POD were observed through the color-coded live body flow images provided. The quantification of blood flow was performed via perfusion units, and then the blood flow was measured via moorLDI Review software (version 6.1). The scanning and analysis of all rats were carried out 3 times, and then the mean value was taken to further evaluate them statistically.

### 2.8. Flap Angiography

On the 7th day after the operation, rats were randomly selected (*n* = 6) to undergo systemic angiography for the evaluation of the microvessel level [32]. An indwelling needle was used for the puncturing of the common carotid artery, followed by quickly injecting lead oxide-gelatin (80 mL per kg of body weight) through a silicone rubber catheter until the skin of the rat's limbs turned yellow. Then, the injected rats were kept at 4°C for fixation (overnight incubation), and the flaps were cut out the next day and filmed with an X-ray machine (54 kVp, 40 mA, 100 s exposure). The vascular J plug-in of ImageJ 1.51 was employed for the calculation of the average microvessel density [33].

### 2.9. Hematoxylin and Eosin (H&E) Staining

On the seventh day postsurgery, the rats were euthanized with an excess dose of sodium pentobarbital, followed by taking six tissue specimens of 1 cm × 1 cm from the flap II area (central tissue). The specimens were then fixed with paraformaldehyde (4%) for 24 h followed by embedding the samples in paraffin, and then the paraffin was cut to a thickness of 4 *μ*m using a microtome. For H&E staining, the sections were fixed on glass slides coated with poly-L-lysine, followed by randomly selecting the 6 areas from the random sections that were found under an optical microscope. Furthermore, the number of microvessels per unit area (per mm^2^) was measured as a measurement index of microvessel density (MVD).

### 2.10. Immunohistochemistry (IHC)

The deparaffinization of the 6 paraffin sections in H&E staining was carried out with the help of xylene, while the rehydration of the samples was carried out via a graded series of ethanol. After washing, endogenous peroxidase activity was inhibited using a 3% H_2_O_2_ solution. Next, the slices were exposed to sodium citrate buffer (10.2 mM) at 95°C for 20 min, followed by blocking with 10% (*w*/*v*) BSA phosphate buffer for 10 min. Finally, slices with primary antibodies, i.e., CD34 (1 : 100), cadherin 5 (1 : 200), VEGF (1 : 200), SOD1 (1 : 100), CTSD (1 : 100), and CASP3 (1 : 200), were incubated overnight at 4°C. Next, slices were exposed to secondary antibodies for one hour at approximately 25°C, reverse stained with hematoxylin, followed by staining the nucleus with DAPI (Beyotime Biotechnology, China). The DP2-TWAIN image acquisition system was employed to image the flap tissue at 200 times magnification. Image-Pro Plus (Media Cybernetics) was used for the measurement of CASP3, VEGF, SOD1, CTSD, and cadherin 5. The overall absorbance of CD34-positive blood vessels was recorded. In total, six random areas in 3 random sections were involved in the IHC analysis.

### 2.11. Immunofluorescence Staining

According to the experimental procedure in the IHC, 6 central organizations were taken from each group II area. Washing and rehydration of the samples were carried out via a graded series of ethanol, while xylene was used to dewax the samples. Next, tissue antigen was extracted with 3% (*v*/*v*) hydrogen peroxide solution and 10.2 mm sodium citrate buffer (20 min, 95°C), and then 0.1% (*v*/*v*) PBS-Triton X-100 was applied to permeate the sample for 30 min. After blocking with 10% (*v*/*v*) PBS for 1 hr, this was followed by incubating with anti-LC3II (1 : 200) and p62 (1 : 100) primary antibodies at 4°C overnight. Next, the cells were reincubated with the secondary antibody at ~25°C for one hour, followed by staining the nuclei with DAPI (Beyotime Biotechnology, China). The images were assessed under a fluorescence microscope. From 3 random sections of each specimen, 6 fields were randomly selected for observation and evaluation of the dermis, and the percentages of LC3II-positive cells and p62-positive cells were calculated.

### 2.12. Immunoblotting

At POD7, after the rats were sacrificed, six 0.5 cm × 0.5 cm specimens were taken from the central tissue of the flaps in zone II of each group and kept at -80°C for immunoblotting and immunoprecipitation analysis. After the skin flap tissue was extracted, its protein content was evaluated via a BCA protein detection kit. Protein separation was carried out via PAGE (12%), followed by transferring onto the PVDF membrane. Then, nonfat milk (5%, *w*/*v*) was used for membrane blockage for 2 h at ~25°C, followed by membrane incubation with the underlying primary antibodies (for 24 h at 4°C): MMP9 (1 : 1,000), VEGF (1 : 1,000), HO1 (1 : 1,000), cadherin 5 (1 : 1,000), SOD1 (1 : 1,000), Bax (1 : 1000), eNOS (1 : 1,000), caspase 3 (CASP3) (1 : 1,000), CYC (1 : 1,000), p62 (1 : 1,000), LC3 (1 : 500), Beclin 1 (1 : 1,000), CTSD (1 : 1,000), VPS34 (1 : 1,000), and GAPDH (1 : 1,000). Then, membrane incubation was carried out with horseradish peroxidase- (HRP-) coupled IgG secondary antibody (1 : 5000) for 2 h at 25°C. The band was visually analyzed via the ECL Plus kit, and the quantification of band intensity was carried out via Image Lab 3.0 software (Bio-Rad, Hercules, CA, USA).

### 2.13. Statistical Analysis

All statistical analyses were performed via the SPSS statistical software package (version 22.0; IL, USA). The obtained results were represented as mean ± SE, and the Wilcoxon test corrected by Benjamini and Hochberg (BH) was used for pairwise comparison. One-way analysis of variance was employed to compare the variations between two groups, and the independent sample *t*-test was employed to compare the two groups. *P* value < 0.05 is regarded as considerable, and the variations are statistically considerable.

## 3. Results

### 3.1. Impact of GAS on the Vitality of Random-Pattern Skin Flaps

**Figure 2:**
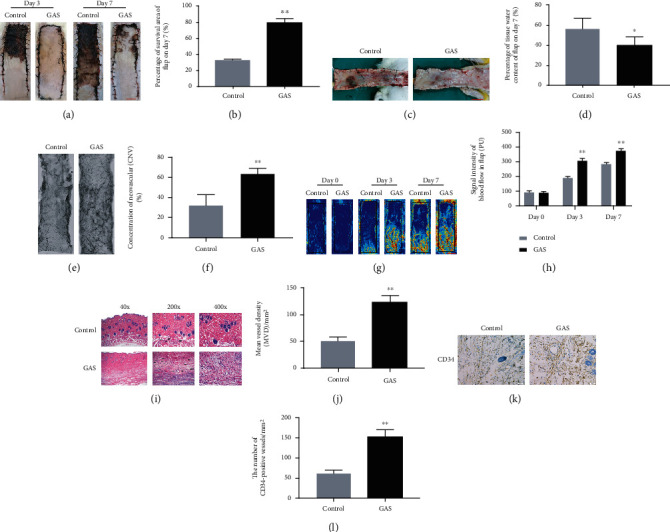
Impact of GAS on the vitality of random-pattern skin flaps. (a) In the control group and the GAS group, the digital photographs of the skin flaps have been taken on the third and seventh day (scale bar: 1.0 cm), respectively. (b) Histogram of the survival area percentage on the seventh day after surgery. (c) Digital photographs (scale bar: 1.0 cm) of tissue edema on the 7th day after surgery in the control group and the GAS group. (d) Histogram of water content percentage in the skin flap tissue. (e) The flap angiography of the 7th PODs in the control group and the GAS group revealed that the blood vessels in the necrotic area were in mass. (f) Measurement of the relevant average microvessel density. There are significant differences between these groups. (g) LDBF imaging shows the blood flow and blood supply in the random-pattern flaps of the control group as well as the GAS group at 0, 3rd, and 7th PODS, respectively (scale bar: 1.0 cm). (h) Representation of the histogram of the blood flow signal intensity of the flap at the 0, 3rd, and 7th days after surgery. (i) H&E staining of microvessels in area II of the flap (original magnification: 200x; scale bar: 50 *μ*m). (j) H&E-stained MVD histogram. (k) CD34 IHC in zone II of the flap (original magnification: 200x; scale bar: 50 *μ*m). (l) Histogram of the percentile of CD34-positive vessel density. The obtained results are represented as mean ± SE, *n* = 6 per group. ^∗^*P* value < 0.05 and ^∗∗^*P* value < 0.01 vs. the control group.

Each rat survived after the flap operation, and no infection was noticed postsurgery. On the 3rd day postsurgery, necrosis occurred in area III of the skin flap in the control group, with lesions (dark brown), and it had the potential for spreading into area II, while area III looked pale and swollen without clear necrosis in the GAS group, as depicted in Figure 2(a). As time progresses, the necrotic area of the flap slowly dries out, the color darkens, and the shrinkage of tissue occurs. The boundary between survival and necrosis progressively expands from the top of the flap to the pedicle, and then stabilizes on the 7th day postsurgery, as depicted in Figure 2(a). The survival area in the GAS group was considerably elevated compared to the control group, as depicted in Figure 2(b). The skin flap tissues were compared in the control as well as in the GAS group. According to the obtained results, edema and venous congestion (subcutaneous) were observed in the control group compared to the GAS group in which mild symptoms of the underlying complications were observed (Figure 2(c)). Quantitatively, the water content of the tissue was lowered in the GAS group, as shown in Figure 2(d). On the 7th day after surgery, neovascularization was observed in the GAS group [34], and also dense vascular clusters were observed in areas I and II and diverged from area III, as depicted in Figure 2(e). In the control group, the new blood vessels were found to be significantly lowered in zone I, and other parts of the vascular system showed complete or incomplete masses (Figure 2(e)). Neovascularization was considerably lowered in the flaps of the control group compared to the GAS group, as depicted in Figure 2(f). The obtained results of laser Doppler indicated the blood flow in the flap and reconstruction of the microvascular network (Figure 2(g)). The results that were analyzed immediately after surgery revealed no considerable variations between the two groups. On the 3rd and 7th day postsurgery, the stronger blood flow signal intensity was observed in both groups, as depicted in Figure 2(h). On the 7th day after surgery, H&E staining revealed a significantly high number of microvessels in the GAS group compared to the control group (Figure 2(i)), and the average vessel density (MVD) was considerably elevated compared to the control group, as depicted in Figure 2(j). In the current study, CD34 IHC was used for the labeling of vascular endothelial cells for quantification of MVD. The results of immunohistochemistry revealed that the CD34-positive blood vessels were elevated in the GAS group compared to the control group (Figures 2(k) and 2(l)). Simultaneously, there were no considerable variations between the GAS group and the control group in the above tests. In conclusion, the underlying results reveal that gastrodin can enhance the flap's survival rate.

### 3.2. GAS Enhances Angiogenesis in Random-Pattern Skin Flaps

**Figure 3:**
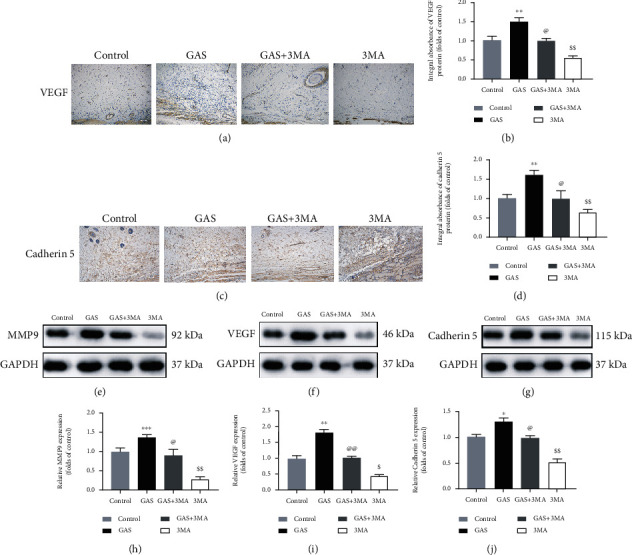
GAS enhances angiogenesis in random-pattern skin flaps. (a, c) IHC expression of VEGF as well as cadherin 5 in skin flaps of the control group, the GAS group, the GAS+3MA group, and the 3MA group (original magnification: 200x; scale bar: 50 *μ*m). (b, d) The histogram of the OD values of cadherin 5 and VEGF was detected via immunohistochemistry. (e–g) Immunoblotting was used to detect VEGF, MMP9, and cadherin 5 in the skin flaps of the control group, the GAS group, the GAS+3MA group, and the 3MA group; GAPDH has been used as an internal control. Gel electrophoresis has been carried out under similar conditions in the experiment. (h–j) Histograms of OD values for VEGF, MMP9, and cadherin 5 as evaluated via immune blotting. The obtained results are expressed as mean ± SE, *n* = 6 per group. ^∗^*P* value < 0.05 and ^∗∗^*P* value < 0.01 vs. the control group.

The blood supply determined by angiogenesis is a significant factor that influences the survival of the flap. So, we explored whether the positive effect of gastrodin on flap survival may be obtained via enhancing angiogenesis. IHC was employed for the detection of cadherin 5 and VEGF expression. VEGF expression was considerably elevated in the flap II area of the GAS group compared to the control group and the GAS+3MA group, while VEGF expression in the 3MA group was significantly lowered, as depicted in Figures 3(a) and 3(b). The results showed consistency with the results obtained from immunoblotting (Figures 3(f) and 3(i)). In the GAS group, the cadherin 5 expression in endothelial and mesenchymal cells was elevated compared to the control group and the GAS+3MA group, while the cadherin 5 expression in the 3MA group was very lowered, as depicted in Figures 3(c), 3(d), 3(g), and 3(j). Furthermore, immunoblotting was employed for the detection of the MMP9 level. Similar to VEGF and cadherin 5 results, the MMP9 expression was considerably elevated in the GAS group compared to the control group and the GAS+3MA group (Figures 3(e) and 3(h)). So, the underlying results revealed that gastrodin enhances angiogenesis in skin flaps.

### 3.3. GAS Inhibits the Apoptotic Process in Random-Pattern Skin Flaps

**Figure 4:**
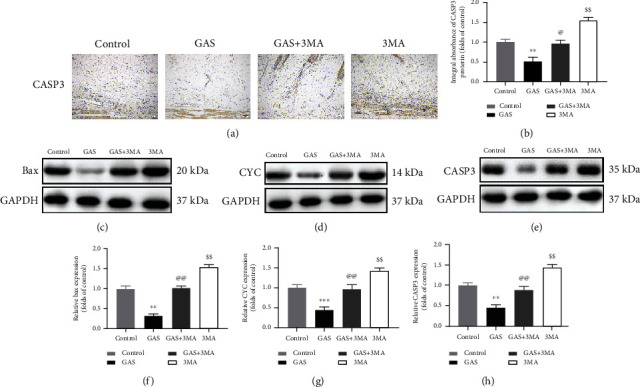
GAS attenuates the apoptosis in skin flaps (random pattern). (a) The CASP3 expression in the flaps of the control, GAS, GAS+3MA, and 3MA groups, using immunohistochemical methods (original magnification: 200x; scale bar: 50 *μ*m). (b) The histogram of the OD value of the expression level of caspase 3 was detected via immunohistochemistry. (c–e) Immunoblotting has been used to determine the CYC, Bax, and caspase 3 expressions; GAPDH has been used as an internal control. Gel electrophoresis has been carried out under similar conditions in the experiment. Western blotting measures the optical density value. (f–h) Histograms of the OD values of VEGF, MMP9, and cadherin 5 in the two groups as evaluated via immunoblotting. The obtained results are expressed as mean ± SE, *n* = 6 per group. ^∗^*P* value < 0.05 and ^∗∗^*P* value < 0.01 vs. the control group.

The impact of GAS was studied on the apoptotic process of random flaps. IHC staining and immunoblotting were employed to determine the expression of proteins associated with apoptosis. IHC and integrated absorbance analysis indicated that the CASP3 expression in the GAS group was considerably decreased compared to the control group and the GAS+3MA group. The CASP3 expression was considerably elevated in the 3MA group; however, no considerable variations were observed between the control group and the GAS+3MA group, as depicted in Figures 4(a) and 4(b). The underlying results showed consistency with the results of immunoblotting (Figures 4(e) and 4(h)). Furthermore, immunoblotting was employed to determine the Bax and CYC expression. The results showed that the Bax and CYC level was considerably decreased in the GAS group compared to the control group and the GAS+3MA group, while no considerable variations were observed between the control group and the GAS+3MA group, as depicted in Figures 4(c), 4(d), 4(f), and 4(g). In conclusion, the underlying results validated that gastrodin significantly contributes in decreasing the apoptotic rate of the cell.

### 3.4. GAS Decreases Oxidative Stress in Random-Pattern Skin Flaps

**Figure 5:**
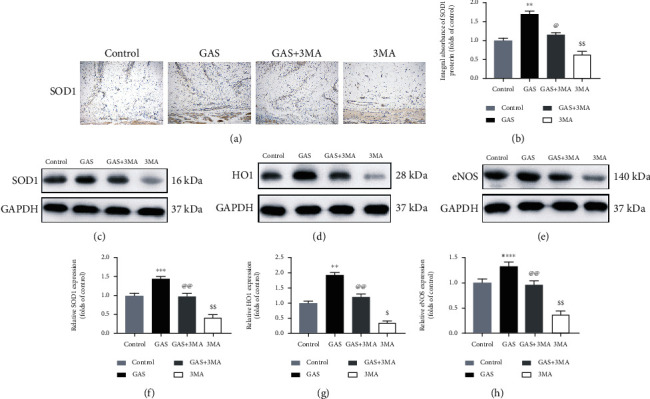
GAS reduces oxidative stress in skin flaps (random pattern). (a) IHC has been employed to determine the expression of SOD1 in the flaps of all groups (original magnification: 200x; scale bar: 50 *μ*m). (b) The histogram of the OD value of SOD1 in each group of IHC. (c–e) Immunoblotting detects the expression of eNOS, SOD1, and HO1 proteins in the flaps. Gel electrophoresis has been carried out under similar experimental conditions, and the sheared spots are indicated here. (f–h) Histograms of the OD values of VEGF, MMP9, and cadherin 5 in all groups as analyzed via immunoblotting. The obtained results are expressed as mean ± SE, *n* = 6 per group. ^∗^*P* value < 0.05 and ^∗∗^*P* value < 0.01 vs. the control group.

The oxidative stress extremely affects the skin flap survival. We have been exploring whether gastrodin regulates oxidative stress. IHC and immunoblotting were employed for the detection of the SOD1 expression in the dermis to determine the oxidative stress level. The obtained results revealed that the SOD1 expression was considerably elevated in the GAS group compared to the control group and the GAS+3MA group (Figures 5(a), 5(b), 5(c), and 5(f)). The levels of HO1 and eNOS protein were elevated in the GAS group, as compared with the other three groups, as shown in Figures 5(d), 5(e), 5(g), and 5(h). However, the underlying results revealed that no considerable variation was observed between the control group and the GAS+3MA group, while the lowest expression was observed in the 3MA group. The underlying results revealed that gastrodin is likely to enhance the survival of skin flaps by decreasing oxidative stress.

### 3.5. GAS Stimulates Increased Autophagy Flux in Random-Pattern Skin Flaps

**Figure 6:**
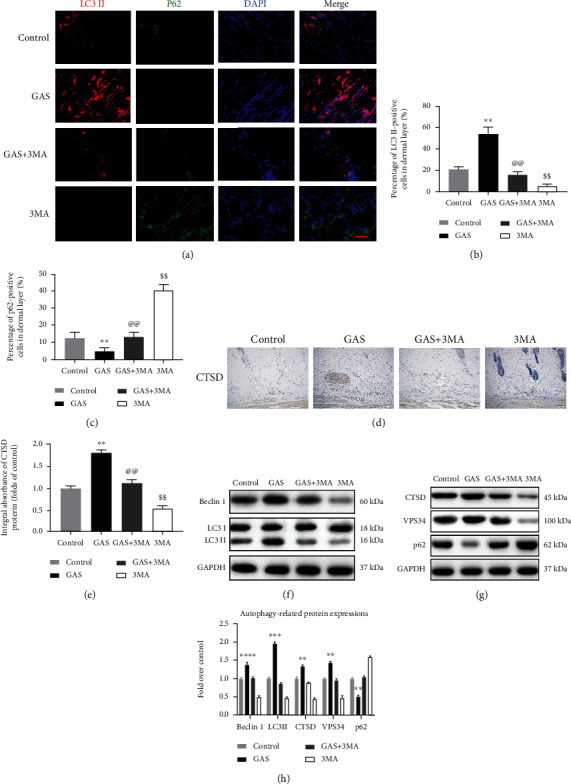
GAS stimulates increased autophagy flux in skin flaps (random pattern). (a) The p62 and LC3II expressions were determined via immunofluorescence which revealed the autophagosomes in the skin flap cells: autophagosomes in the dermis of zone II (red); p62-positive cells (green); and the nucleus, which was stained with DAPI (blue) (scale bar: 50 mm). (b, c) Histogram of the percentages of p62-positive and LC3II-positive cells. (d) The IHC method was used to detect the CTSD level of skin flaps in all groups (original magnification: 200x; scale bar: 50 *μ*m). (e) Histogram of IHC CTSD OD values in each group. (f, g) Immunoblotting of LC3II, Beclin 1, Vps34, CTSD, and p62 proteins in the control group, the GAS group, the GAS+3MA group, and the 3MA group. Gel electrophoresis was carried out under similar conditions in the experiment, and the sheared spots are indicated here. (h) Histograms of the OD values of LC3II, Beclin 1, VPS34, CTSD, and p62 in the two groups as identified via immunoblotting. The obtained results are expressed as mean ± SE, *n* = 6 per group. ^∗^*P* value < 0.05 and ^∗∗^*P* value < 0.01 vs. the control group.

In the existing study, to analyze how gastrodin stimulates the autophagy of random flaps and increase the autophagic flux, we evaluated Vps34, Beclin1, and LC3II protein which are the main constituents of autophagosomes, while the autophagy substrate protein SQSTM1/p62 is used for the detection of autophagy flux and the autophagolysosome representative protein CTSD. Immunofluorescence was employed to determine the frequency of LC3II and p62-positive cells in the dermis (Figure 6(a)). The obtained results revealed that the autophagosomes were significantly activated in the GAS group compared to the control group; the frequency of LC3II-positive spots was considerably elevated, while the frequency of p62-positive spots showed opposite results (Figure 6(a)). The use of the autophagy inhibitor 3MA considerably attenuated the underlying process, as shown in Figures 6(b) and 6(c). The obtained results of IHC analysis revealed that the CTSD level was elevated in the GAS group (Figures 6(d) and 6(e)), which was also validated via immunoblotting (Figures 6(g) and 6(h)). Immunoblotting results also revealed that the autophagy proteins, i.e., Vps34, Beclin 1, and LC3II, were upregulated in the GAS group, while the p62 expression was considerably lowered in the GAS group compared to the control group (Figures 6(f), 6(g), and 6(h)). The results of the GAS+3MA group showed similarity with the control group. Furthermore, the results of IHC and immunoblotting revealed that 3MA treatment decreased the CTSD expression (Figures 6(d), 6(e), 6(g), and 6(h)). Western blotting was further used for the detection of the Vps34, Beclin1, p62, and LC3II expression. The obtained results showed that post 3MA treatment, the expression level of p62 in the flap tissue was elevated, while the expression levels of Beclin1, LC3II, and Vps34 were lower (Figures 6(f), 6(g), and 6(h)). The underlying results validated that gastrodin can indeed stimulate the increase of autophagy flux in the skin flap, which may be the reason for the previously observed inhibition of oxidative stress, reduced apoptotic process, and elevated angiogenesis. In brief, GAS can stimulate autophagy, while 3MA can attenuate GAS-activated autophagy.

### 3.6. Attenuation of Autophagy Reverses the Effects of GAS on the Apoptotic Process, Oxidative Stress, and Angiogenesis and Depresses the Survival of Random-Pattern Skin Flaps

**Figure 7:**
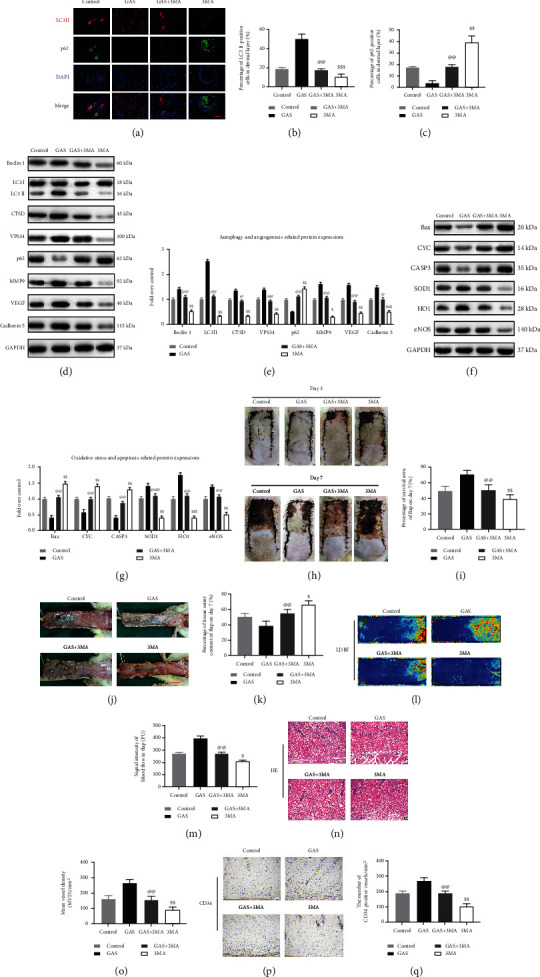
Attenuation of autophagy reverses the impact of GAS on oxidative stress, apoptotic process, angiogenesis, and the survival of the flap. (a) Immunofluorescence staining showed that there were autophagosomes (red) in the cells of the flaps in the control group, the GAS group, the GAS+3MA group, and the 3MA group via LC3II (scale bar: 20 *μ*m). p62-positive cells (green) (b, c). The percentages of LC3II-positive cells and p62-positive cells were quantitatively analyzed in the dermis of all groups. (d, f) Western blotting was used to detect autophagy-associated proteins, i.e., Beclin1, Vps34, SQSTM1/p62, CTSD, and LC3II; angiogenesis-associated proteins, i.e., VEGF, MMP9, and cadherin 5; apoptosis-associated proteins, i.e., CYC, Bax, and CASP3; and oxidative stress-associated protein, i.e., HO1, SOD1, and eNOS in all groups. Gel electrophoresis was carried out under the same conditions in the experiment, and the cropped spots are indicated here. (e, g) The OD values of Beclin1, Vps34, SQSTM1/p62, CTSD, MMP9, LC3II, cadherin 5, VEGF, CYC, CASP3, Bax, HO1, eNOS, and SOD1, in all groups. (h) Digital photos (scale bar: 1 cm) of the flaps in the control group, the GAS group, the GAS+3MA group, and the 3MA group on POD3 and POD7. (i) Quantitative analysis of the percentage of survival area in each group. (j) Digital photos have been taken from inside of the flaps in the control group, the GAS group, the GAS+3MA group, and the 3MA group on POD7 (scale bar: 1 cm). (k) Histogram of the percentage of water content in each group. (l) Full-field LDBF image of each group of skin flaps in POD7 (scale bar: 1 cm). (m) Quantitative analysis of the blood flow signal intensity of skin flaps in each group. (n) H&E staining shows the blood vessels in the flap zone II of the control group, the GAS group, the GAS+3MA group, and the 3MA group (original magnification: 200x; scale bar: 50 *μ*m). (o) Histogram of MVD percentage in each group. (p) CD34IHC in the control group, the GAS group, the GAS+3MA group, and the 3MA group showed the blood vessels in zone II (original magnification: 200x; scale bar: 50 *μ*m). (q) Histogram of the percentage of CD34-positive blood vessels in all groups. The obtained results are represented as mean ± SE, *n* = 6 for all groups. ^$^*P* value < 0.05 and ^$$^*P* value < 0.01, in comparison with the control group; ^@^*P* value < 0.05 and ^@@^*P* value < 0.01, relative to the GAS group.

GAS can stimulate autophagy, and to confirm its mechanism, the efficacy of the 3MA group and the GAS+3MA group was evaluated, accordingly. The obtained results revealed that in the GAS+3MA group, the frequency of the LC3II-positive cells in the flap dermis was decreased compared to the GAS group, while an increasing rate of p62-positive cells was observed. Simultaneously, the average frequency of LC3II in the 3MA group was decreased compared to the control group, while the average frequency of p62 was elevated compared to the control group, as depicted in Figures 7(a), 7(b), and 7(c). According to immunoblotting that compared the GAS group, the SQSTM1/p62 expression was elevated and the expression of Vps34, CTSD, Beclin 1, and LC3II was decreased in the GAS+3MA group, as depicted in Figures 7(d) and 7(e). The underlying results revealed that 3MA treatment increases the SQSTM1/p62 expression while it decreases the expression of Vps34, Beclin1, LC3II, and CTSD (Figures 7(d) and 7(e)). In summary, the underlying results indicated that in the flap model, 3MA inhibits the effect of gastrodin to stimulate the increase of autophagic flux. The obtained results revealed that the MMP9, VEGF, and cadherin 5 expression was considerably decreased in the GAS+3MA group of rats compared to the GAS group, while the expression of underlying proteins was considerably reduced in the 3MA group of rats compared to the control group, as depicted in Figures 7(d) and 7(e). Compared with the GAS group, the expression of SOD1, eNOS, and HO1 was considerably decreased in the GAS+3MA group, while the expression of underlying enzymes was also reduced in the 3MA group compared to the control group (Figures 7(f) and 7(g)). In contrast, the expression levels of CASP3, BAX, and CYC were considerably elevated in the GAS+3MA group compared to the GAS group, and their expression in the 3MA group was also considerably elevated compared to the control group (Figures 7(d)–7(g)). These results suggested that protein regulation was significantly affected by 3MA when used in combination with gastrodin. Furthermore, we also explored whether the therapeutic benefits of gastrodin in flap survival, edema, and angiogenesis will be eliminated or affected by the combined application of 3MA. In the control, GAS, GAS+3MA, and 3MA groups, no considerable variations were observed in the flap survival rate on the third day postsurgery. However, on the seventh day, the flap survival rate of the 3MA group was considerably decreased compared to the control group, and the flap survival rate of the GAS+3MA group was considerably decreased compared to the GAS group, while the degree of edema and subcutaneous venous congestion increased significantly (Figures 7(h)–7(j)). The water content of the tissue in the 3MA group was elevated compared to the control, GAS+3MA, and GAS groups, as depicted in Figure 7(k). LDBF results revealed that 3MA could reduce the blood flow of the flap, as compared with the control. It has also been observed that the blood flow was reduced by the combined effect of GAS+3MA, as compared with the use of GAS alone, as depicted in Figures 7(l) and 7(m). H&E staining indicated that the microvessels were lowered in the 3MA group as well as in GAS+3MA group (Figure 7(n)), and the average blood vessel density was considerably decreased compared to the control group and the GAS group (Figure 7(o)). At the same time, IHC showed fewer CD34-positive blood vessels (Figures 7(p) and 7(q)). In summary, the present study revealed that 3MA eliminates the effect of GAS to stimulate autophagy flux, affects the regulation of GAS on key proteins, and also impairs the positive role of gastrodin in skin flap treatment.

## 4. Discussion

GAS is the main ingredient of the Chinese herbal medicine *Gastrodia elata*. It is well known for its significant activity against inflammation [35]. For many decades, it has widely been used in the research study of dizziness and epilepsy [36]. GAS not only contributes to the protection of the nervous system but also has an effective role in the treatment of other disease models [37]. It has also been reported that GAS can lower the apoptotic process of endothelial cells and enhance the functions of endothelial cells caused by oxidative stress through stimulation of the Nrf2/HO1 cascade. The underlying study reveals that GAS significantly contributes to wound healing [38]. In the rat osteoarthritis model, GAS also has a protective role in chondrocyte apoptosis stimulated via IL-1*β*. Furthermore, GAS suppresses the NF-*κ*B (nuclear factor-*κ*B) cascade, decreases the release of inflammatory mediators (IL-6*α*), and reduces the catabolism of the chondrocyte matrix induced by IL-1*β*. GAS can enhance the degeneration of cartilage in the *in vivo* knee OA model of rats and has the potential as a candidate therapeutic drug for OA [39]. Moreover, few studies have been reported on the role of GAS in stimulating the increase in autophagy flux, particularly in the skin flap model. In the current study, we selected the optimal concentration of gastrodin and 3MA on the basis of a previous study and gave IP injections [32, 35]. The obtained results reveal that GAS can stimulate the increase of autophagy flux, induce angiogenesis, decrease the oxidative stress and apoptotic process, and promote the survival of random-pattern skin flaps. These findings demonstrate the clinical potential of GAS to improve the survival of random-pattern skin flaps.

A random-pattern flap is a tool that is used in reconstructive and plastic surgery, and the survival area of the flap has a strong correlation with the angiogenesis after the formation of the flap. Earlier studies have revealed that enhancing angiogenesis can effectively improve the random-pattern flap survival [11, 40]. In the current study, the administration of GAS treatment significantly increased the number of microvessels in the dermis of random flaps and enhanced the flow of blood in the flaps. Angiogenesis is an extremely complicated process that involves mitosis, germination, growth and development, migration of endothelial cells, and the development of new capillaries, connecting with preexisting cells to form new blood vessels [41–43]. It has been reported that VEGF acts on endothelial cells and binds with the tyrosine kinase receptor, i.e., VEGFR2, which is a cell surface receptor, and initiates a series of signaling cascades through PI3K and MAPK pathways, thus enhancing the migration of endothelial cells, proliferation, and angiogenesis, while also elevating the microvascular permeability [41, 44]. MMP9 improves the remodeling of tissue through degradation of the extracellular matrix, and it has a significant role in the release of VEGF and angiogenesis [45, 46]. In addition, cadherin 5 is particularly expressed in the adhesion junctions of endothelial cells and significantly contributes to intercellular adhesion and signal cascades, preventing new blood vessels from decomposing into disorganized endothelial aggregates [47]. The underlying results revealed that after treatment with gastrodin, the expression levels of VEGF, MMP9, and cadherin 5 were considerably elevated in the dermal blood vessels and stromal cells. In summary, GAS promotes neovascularization by increasing the levels of MMP9, VEGF, and cadherin 5, and elevating the flap survival rates.

Tissue ischemia is the most common problem after the establishment of a skin flap. With the restoration of blood supply, the tissue faces IRI again, and a high level of ROS is generated, which is the main cause of partial or complete necrosis of the skin flap [48, 49]. In the reperfusion phase, the accumulated ROS reacts with lipids and proteins of the cell membrane, which leads to cell membrane destruction by destroying the cell membrane through lipid peroxidation [50]. Then, the underlying process can also lead to the destruction of nucleic acids and chromosomes and activate oxidative stress and cellular apoptosis [51]. As an antioxidant enzyme, SOD is the first defense system of cells against oxidative stress damage [52]. HO1 and eNOS can be expressed in response to oxidative stress [53, 54]. It has been reported that GAS can significantly enhance the protection of endothelial fibrosis and promote wound healing by reducing endothelial cell apoptosis induced by oxidative stress [38]. The results obtained from immunoblotting and IHC revealed that gastrodin can elevate the eNOS, SOD1, and HO1 expression in the dermis of ischemic skin flaps. The underlying results revealed the antioxidant properties of gastrodin and its protective effect on IRI.

According to reports, gastrodin decreases inflammation induced via IL-1*β* and related apoptosis of chondrocytes (rat) via attenuating the NF-*κ*B signaling pathway cascade. *In vivo* studies have also validated the antiapoptotic effects of gastrodin [39]. Apoptosis is a highly conservative process, which is associated with many physiological and pathological conditions, i.e., an ischemia-reperfusion injury that may result in the necrosis of a skin flap. Apoptosis might be enhanced by internal (mitochondrial damage or ROS) or exogenous (by growth factors) factors. Mitochondria are in the central position during apoptosis. ROS quickly enhances the inner mitochondrial membrane permeability. BAX induces the permeability of the outer mitochondrial membrane, leading to swelling. CYC has been released from mitochondria that form apoptotic bodies, which leads to the activation of caspase 3 to induce apoptosis [55, 56]. Therefore, the degree of apoptosis can be analyzed by measuring the levels of CYC, Bax, and CASP3. Our data indicated that gastrodin attenuates the expression of apoptosis-associated proteins, i.e., CYC, Bax, and CASP3. The underlying results revealed that GAS can inhibit the apoptosis of random-pattern flaps, which is beneficial to the survival of the flaps.

Macroautophagy (hereafter named autophagy) is a highly conservative cellular self-degradation process. In the underlying process, the poisons, unnecessary proteins, and organelles have been degraded in the cell via the autophagy-lysosome cascade, and play a significant role in the survival and maintenance of cells [57]. The autophagy flux is defined as the autophagy process of lysosomal proteases from autophagosome formation to cargo transportation and lysosome degradation [57]. In vascular diseases, autophagy plays a significant role. In a mouse model of chronic ischemic remodeling, autophagy has a protective role in the angiogenesis of endothelial progenitor cells and decreases cellular apoptosis, myocardial hypertrophy, and fibrosis [58]. Earlier studies have revealed that the stimulation of autophagy can enhance the survival of the random-pattern flap [20, 40]. Furthermore, it has been reported that gastrodin pretreatment can reduce myocardial ischemia/reperfusion injury by promoting autophagy flux [59], which reveals that GAS has significant effects in the random-pattern flaps. However, there is a lack of clarity regarding the correlation between gastrodin and autophagy in random-pattern flaps. To the best of our knowledge, this is the first time we have studied the effect of GAS on autophagy in random-pattern flaps. The process of autophagy has been well studied which includes three consecutive steps: isolation, transportation, and degradation. The Vps34, Beclin1, and LC3II levels have been elevated in the isolation phase, and during the process of transportation and degradation, the autophagic flux has been stimulated that results in the depletion of SQSTM1/p62 [60, 61]. CTSD is a lysosomal aspartic protease that significantly contributes to the degradation stage [62]. In the present study, immunofluorescence and IHC results showed that in the GAS group the levels of LC3II and CTSD were considerably elevated in the dermis of the flaps, while p62 expression was reduced in the underlying group. Immunoblotting results revealed that the Vps34, Beclin1, and LC3II levels were also elevated in the GAS group compared to the control group, suggesting that additional autophagosomes were formed in the random-pattern flap. In addition, CTSD expression was elevated in the flaps of the GAS group, while p62 protein level was considerably decreased in the underlying group, which suggests that the autophagic flux was elevated in the GAS group. The above results indicate that GAS enhances the autophagy of random-pattern flaps and stimulates the increase of autophagy flux.

Autophagy plays a double-edged sword in the survival of cells. In many diseases, the stimulation of autophagy is conducive to the survival of cells, such as cardiovascular diseases [63]. On the other hand, overactivated autophagy might activate apoptosis [64]. So, we also evaluated the role of GAS-induced autophagy in random-pattern flaps. 3MA is the most common inhibitor of autophagy, which can affect the formation of autophagosomes in eukaryotic cells by inhibiting PI3K [65]. Suppression of autophagy via 3MA reversed the effects of GAS-activated elevation in the survival of a flap, decreased tissue edema, and elevated microvessel density. Autophagy can promote angiogenesis via triggering the phosphorylation of AKT in endothelial cells [66]. Our results showed that 3MA considerably decreased the levels of MMP9, VEGF, and cadherin 5. So, GAS enhances the angiogenesis of random-pattern flaps via stimulating autophagy. Previous studies on intervertebral disc degeneration have shown that activated autophagy can reduce mitochondrial dysfunction and cellular apoptosis under oxidative stress [18]. Our data showed that after 3MA treatment, the expression of apoptosis-associated proteins, i.e., CYC, Bax, and CASP3, in the dermis of random-pattern flaps increased significantly, while the oxidative stress-associated proteins, i.e., HO1, eNOS, and SOD1, decreased significantly. The underlying variations revealed that GAS attenuates cellular apoptosis and decreases oxidative stress via stimulating autophagy.

Taken together, our results revealed that after GAS treatment, the Vps34, Beclin 1, CTSD, and LC3II levels were elevated in the flap, while the SQSTM1/p62 level was lowered in the flap, which suggested that GAS stimulates the increase of autophagy flux. In addition, 3MA, an effective inhibitor of autophagy, reversed the therapeutic benefits of GAS on the survival of a flap and its prognosis, indicating that autophagy is the reason for the remarkable effects of GAS on flaps.

## 5. Conclusions

In conclusion, the present research work revealed that GAS activates autophagy; promotes the increase of autophagy flux, promoting angiogenesis; and attenuates apoptosis and oxidative stress; and it can also improve the survival of the random-pattern flap.

## Data Availability

The original data supporting the conclusions of this article will be provided by the corresponding author to any qualified researcher without reservation.
